# A Protein-Based Blood Test for Multi-Cancer Diagnostics

**DOI:** 10.3390/biomedicines13102510

**Published:** 2025-10-15

**Authors:** Douglas Held, Steven Bolland, Robert Freese, Robert Puskas

**Affiliations:** 1 Traxxsson, LLC, Saint Louis, MO 63021, USA; 2 Definitive Diagnostics, New York, NY 10021, USA

**Keywords:** multi-cancer detection, protein biomarkers, early diagnosis

## Abstract

**Background/Objectives:** Conventional cancer screening relies heavily on imaging and invasive procedures, leading to high false-positive rates and limited uptake, while leaving several high-mortality cancers without routine screening options. This study evaluated a protein-based multi-cancer early detection (MCED) test designed to detect five high-burden cancers with high sensitivity, specificity, and tissue-of-origin (TOO) accuracy. **Methods:** Serum from 141 patients with confirmed breast, lung, colorectal, ovarian, or pancreatic cancer and 119 healthy controls was analyzed using a 16-parameter protein biomarker panel. The assay measured extracellular protein kinase A (xPKA) activity, additional kinase activities, and cancer-associated antibodies (IgG, IgM). A supervised, rule-based classification framework was developed for cancer detection and TOO assignment. **Results:** The MCED test achieved 100% sensitivity across all five cancer types and 97% overall specificity, with 98% TOO accuracy. Importantly, 100% of Stage I cancers were detected. Cancer specificities ranged from 96.6% (breast) to 100% (ovarian, pancreatic, and colorectal). **Conclusions:** This protein-based MCED approach demonstrates exceptional performance for multi-cancer detection and TOO identification, including robust early-stage detection, and may reduce the downstream diagnostic burden relative to the existing system.

## 1. Introduction

Cancer screening programs face substantial challenges that limit their effectiveness in reducing mortality. Current screening modalities depend heavily on imaging technologies with inherent limitations. Mammography screening generates false-positive results in approximately 10% of cases, with 95% of these being false positives requiring additional testing [1]. Similarly, low-dose computed tomography (LD-CT) screening for lung cancer produces false-positive results in 33% of cases [2]. These high false-positive rates contribute to patient anxiety, unnecessary invasive procedures, and increased healthcare costs.

The confirmation process for suspicious findings adds complexity and delays to the diagnosis. Mid-sized pulmonary nodules detected by LD-CT require serial imaging over 6–12 months to assess growth patterns [3], yet aggressive lung cancer subtypes can double in size within three months [4]. Colorectal cancer screening relies on invasive colonoscopy procedures, contributing to suboptimal compliance rates. Furthermore, several high-mortality cancers, including ovarian and pancreatic cancers, lack established screening protocols entirely.

Screening compliance remains problematic across cancer types: only 78% of eligible women receive mammograms, 60% of eligible individuals undergo colonoscopy screening, and fewer than 10% of eligible high-risk individuals receive LD-CT screening at recommended intervals [5,6,7]. Recent recommendations to lower the breast cancer screening age from 45 to 40 years have put further strain on an already overburdened screening infrastructure, particularly affecting underserved rural populations with limited access to specialized imaging facilities.

Despite these challenges, early detection remains crucial for improving patient outcomes. Mammographic screening has contributed to an 11% reduction in female breast cancer mortality, while LD-CT screening reduces lung cancer mortality by 16–24% in high-risk populations [8]. However, breast cancer incidence rates continue to increase, with the steepest increases in women under 50 years, emphasizing the need for accessible screening methods for younger populations.

Liquid biopsy approaches using blood-based biomarkers offer potential solutions to current screening limitations by providing non-invasive, easily accessible testing methods that could improve compliance rates. However, existing multi-cancer detection (MCD) tests face significant limitations. Current commercial MCD tests, including those from Grail (Galleri) and Thrive (CancerSEEK), rely primarily on circulating tumor DNA (ctDNA) or cell-free DNA (cfDNA) detection [9,10,11]. These approaches suffer from low target concentrations, particularly in early-stage disease, resulting in poor sensitivity for early cancer detection when intervention is most effective.

Tissue-of-origin determination presents another significant challenge for existing MCD tests. Grail’s Galleri test fails to provide a definitive TOO identification in approximately 1 in 15 cases, requiring physicians to pursue multiple diagnostic pathways [9]. Thrive’s CancerSEEK demonstrates even higher rates of TOO uncertainty [11]. These limitations necessitate extensive downstream testing, reducing the practical utility of these screening approaches.

Recent advances in protein biomarker research have identified abundant serum proteins that are elevated consistently across multiple cancer types and detectable at early disease stages. Unlike DNA-based markers, protein biomarkers offer advantages including higher concentrations in circulation, direct functional relevance to cancer biology, and more stable detection characteristics. Studies have demonstrated that cancer triggers autoimmune responses that produce detectable antibody signatures [12,13,14,15]. These findings suggest that protein-based approaches may overcome the limitations of current ctDNA-based methods.

The present study was designed to develop and validate a comprehensive protein-based MCED test capable of detecting five major cancers (breast, lung, colorectal, ovarian, and pancreatic) with high sensitivity and specificity, while providing accurate tissue-of-origin identification. Our approach focuses on measuring abundant serum proteins, including extracellular protein kinase activities and cancer-associated antibodies that are consistently different across cancer types and detectable at early disease stages, addressing the critical gap in effective multi-cancer screening technologies.

## 2. Materials and Methods

Serum samples were procured from two established biorepository companies: ProMedDx (Norton, MA, USA) and ProteoGenex (Culver City, CA, USA). All samples were collected with institutional review board approval and with documented informed consent from all participants. The study cohort comprised 141 cancer patients and 119 healthy controls, with cancer samples representing five cancer types: breast (*n* = 61), lung (*n* = 43), colorectal (*n* = 13), ovarian (*n* = 13), and pancreatic (*n* = 11). Sample selection criteria required a confirmed histological diagnosis for cancer cases prior to any treatment and an absence of known malignancy for controls.

The comprehensive biomarker panel included measurement of extracellular protein kinase A (xPKA) activity, additional kinase activities, and cancer-associated antibodies in both IgG and IgM forms, totaling 16 distinct parameters.

Extracellular PKA activity was quantified using the MESACUP Protein Kinase Assay Kit (Medical & Biological Laboratories Co., (Tokyo, Japan) Product code 5230). Briefly, 108 μL of the serum samples were mixed with 12 μL of activating buffer (25 mM KH_2_PO_4_, 5mM EDTA, 150 mM NaCl, 50% glycerol *w*/*v*, 1 mg/mL BSA, and 100 mM DTT, pH 6.5) and incubated at room temperature for 30 min for optimal PKA activation. Activated samples (108 μL) were then combined with 108 μL of kit reaction buffer with or without 0.5 μM protein kinase A inhibitor PKI (Santa Cruz Biotechnology (Santa Cruz, CA, USA) sc-201160).

The reaction mixture was incubated with an immobilized peptide substrate for 30 min at 25 °C with agitation at 750 rpm. Peptide phosphorylation was detected using biotinylated phosphoserine antibodies followed by peroxidase-conjugated streptavidin according to the manufacturer’s protocols. Colorimetric detection employed a TMB substrate (Millipore Sigma, (Saint Louis, MO, USA) T0440) with a 60-min incubation, terminated with 0.2 M H_2_SO_4_ stop solution. Absorbance readings were obtained at 450 nm using bovine PKA catalytic subunit standards to generate activity curves. Net xPKA activity was calculated as follows: Net xPKA = Kinase (0 μM PKI)–Kinase (0.5 μM PKI).

Assay performance characteristics included the following: limit of detection (LoD) 0.3 mU/mL, limit of quantification (LoQ) 0.6 mU/mL, quantification range 0.6–500 mU/mL, and average reproducibility coefficient of variation 3.7%.

Supplementary kinases were measured using an assay analogous to the xPKA assay with appropriate peptide targets. Cancer-associated antibodies were quantified using standard enzyme-linked immunosorbent assay (ELISA) protocols with colorimetric endpoint detection. Both IgG and IgM antibody forms were measured for each cancer-associated protein target to capture innate and activated immune responses.

Statistical outliers were identified using the interquartile range method, where values exceeding 1.5 times the interquartile range beyond the first and third quartiles were flagged for review. Outliers were retained in the analysis unless technical measurement errors were identified.

Biomarker patterns for multi-cancer detection employed a supervised, rule-based analytical strategy analogous to supervised machine learning approaches. Training data comprised biospecimens from patients with confirmed cancer diagnoses and healthy controls, labeled according to their known disease status. The primary objective was to identify biomarker value combinations that robustly differentiated cancer from non-cancer states and distinguished between specific cancer types.

Initial pattern discovery utilized quantitative biomarker distribution analysis through box plots and summary statistics to identify the value ranges selectively present in cancer samples versus controls. Optimal threshold values for each biomarker were established, where separation between groups was maximized.

Cancer-type-specific conditional rules were developed using if-then logic structures. This approach represents a form of rule-based supervised classification where labeled training data inform rule structure and threshold determination. Cross-reactivity between cancer types, where patterns flagged multiple cancer types, was resolved through the incorporation of additional biomarkers not previously utilized in specific patterns or fine-tuning threshold values to improve cancer-type exclusivity.

Finalized rule sets underwent independent validations by qualified biostatisticians using SAS software, confirming that the rule-based system achieved its target sensitivity and specificity parameters across multiple cancer types. An external review replicated the initial pattern identification using labeled data, verifying the approach as a practical application of supervised pattern discovery methodology.

Statistical analysis was performed using SAS Version 9.4 (SAS Institute, Cary, NC, USA). All data underwent standardization for variables, labels, and presentation format to facilitate SAS dataset conversion. For continuous variables, descriptive statistics included sample size (*n*), mean, standard deviation, median, minimum, and maximum values. Categorical variables presented the number and percentage of samples in each demographic and disease category. A cross-validation analysis was conducted using 80–20 data splitting for breast and lung cancer cohorts to assess model robustness and potential overfitting. Performance metrics included sensitivity, specificity, and overall accuracy with 95% confidence intervals calculated using exact binomial methods.

## 3. Results

The study’s cohort demographics are summarized in Table 1. The cancer cohort had a mean age of 59.8 years (range 31–79) compared to 58.5 years for the controls (range 46–75). Cancer distribution by type included the following: breast cancer (*n* = 61, 43.3%), colorectal cancer (*n* = 13, 9.2%), lung cancer (*n* = 43, 30.5%), ovarian cancer (*n* = 13, 9.2%), and pancreatic cancer (*n* = 11, 7.8%). All participants were Caucasian. The gender distribution varied by cancer type, with breast cancer being predominantly female (96.7%) and lung cancer predominantly male (67.4%).

The disease staging analysis revealed a comprehensive representation across cancer stages. Importantly, the cohort included 47 Stage I cancers (33.3% of total cancer cases), with representation across all cancer types: breast (*n* = 14), lung (*n* = 31 Stage IA, IB), colorectal (*n* = 1), and pancreatic (*n* = 1). Advanced-stage disease (Stage III-IV) comprised 29.1% of cases, providing assessment capability across the disease spectrum.

The supervised rule-based classification system successfully identified distinct biomarker patterns for each cancer type. Initial pattern development revealed that 15% of cancer cases exhibited biomarker patterns similar to other cancer types, termed “crossovers.” These crossovers were systematically addressed through rule refinement using additional biomarker parameters and threshold optimization, reducing the crossover incidence to 2% and achieving 98% tissue-of-origin accuracy.

**Figure 1:**
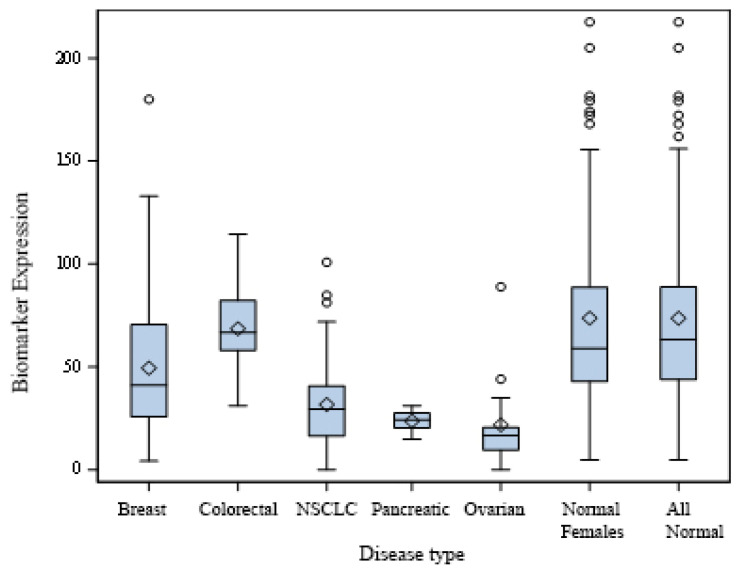
Plot of xPKA values. The length of the box represents the interquartile range (the distance between the 25th and 75th percentiles). The diamond symbol in the box interior represents the group mean. The horizontal line in the box interior represents the group median. The vertical lines (called whiskers) extending from the box show the group’s minimum and maximum values. The empty circles represent outliers.

Figure 1 demonstrates the distribution of extracellular PKA (xPKA) values across cancer types compared to healthy controls. The box plot analysis reveals a clear separation between cancer and control groups for most cancer types. Breast cancer patients showed reduced xPKA levels (median 50 mU/mL) compared to controls (median 75 mU/mL). Similar patterns were observed for lung cancer (median 35 mU/mL), ovarian cancer (median 23 mU/mL), and pancreatic cancer (median 25 mU/mL).

A detailed analysis of individual kinase activities revealed cancer-type-specific changes (Table 2). Lung cancer showed distinct patterns with reduced kinase mean activities for xPKA, Kinase 1, and Kinase 4. Colorectal cancer exhibited elevated levels of Kinase 1 and 4 (mean 210 mU/mL vs. 120 mU/mL and mean 275 mU/mL vs. 215 mU/mL, respectively) and reduced values for Kinase 2 and 3. The mean ovarian cancer values were low for all the kinases. Breast and Pancreatic cancers demonstrated characteristic reductions across all kinases except for Kinase 2.

While specific antibody immunoglobulin classifications (IgM versus IgG) are not individually delineated to preserve the proprietary nature of the biomarker panel, comprehensive antibody measurements encompassing both immunoglobulin classes are presented in Table 2. Cancer-associated antibody measurements indicated that Anti-5 mean values were elevated for colorectal and pancreatic cancers (Table 2). Anti-11 mean or median values were elevated for NSCLC and ovarian cancers. All antibodies showed mean variances for at least one cancer.

Tumors may interfere with B-cell maturation or function, leading to impaired humoral immunity. This can result in fewer circulating autoantibodies despite the presence of tumor-associated antigens [16]. Overall, however, it is the composite of individual values per sample that form the patterns that indicate cancer is present in this study.

The comprehensive MCED test achieved exceptional performance characteristics across all five cancer types (Table 3). Sensitivity reached 100% for each individual cancer type with zero false negatives observed. Specificity varied by cancer type as follows: ovarian (100%), colorectal (99.2%), pancreatic (100%), lung (96.7%), and breast (96.6%), yielding an overall specificity of 97%. PPVs for breast and lung cancer were 64% and 26%, respectively, and NPVs were 100%.

Critically, the test demonstrated 100% sensitivity for Stage I cancer detection across all cancer types. This represents a significant advancement over existing MCD technologies, which show poor sensitivities for early-stage disease. Stage I detection included 14/14 breast cancers (100%) and 31/31 lung cancers (100%).

Ninety-five percent confidence intervals for performance metrics are presented in Table 4. Sensitivity confidence intervals were narrower for breast cancer (95% CI: 94.1–100%) and lung cancer (95% CI: 94.1–100%) due to larger sample sizes. Colorectal, ovarian, and pancreatic cancers showed broader confidence intervals reflecting smaller sample sizes: colorectal sensitivity 95% CI: 75.3–100%, ovarian sensitivity 95% CI: 75.3–100%, and pancreatic sensitivity 95% CI: 71.5–100%. There are multiple reasons why small sample sets have wider confidence intervals than large data sets. The primary reason is that each subject in a small sample set has a much larger influence on the overall estimate of performance than if the sample set were larger.

Specificity confidence intervals demonstrated consistent performance across cancer types, with breast cancer specificity 95% CI: 94.7–100% and lung cancer specificity, 95% CI: 95–100%. Tissue-of-origin accuracy achieved 95% CI: 94.2–99.1% overall.

A cross-validation analysis using 80–20 data splitting was performed for breast and lung cancer. Breast cancer had a mean accuracy of 98% ± 1.5% while lung cancer achieved a mean accuracy of 98.0% ± 1.4%. These results suggest minimal overfitting despite the relatively limited sample size and confirm the model’s generalizability for the two largest cancer cohorts.

## 4. Discussion

This study demonstrates that protein-based biomarker approaches can achieve exceptional performance for multi-cancer detection, addressing critical limitations of current screening methodologies. The achievement of 100% sensitivity across five major cancer types, combined with 97% overall specificity and 98% tissue-of-origin accuracy, represents a significant advancement in liquid biopsy technology for cancer screening applications.

The ability to detect 100% of Stage I cancers across all target cancer types addresses a fundamental requirement for effective cancer screening that current MCD technologies fail to meet. Early-stage detection is crucial for improving patient outcomes, as five-year survival rates for Stage I cancers typically exceed 90% compared to 10–30% for Stage IV disease. This capability could transform cancer screening by enabling intervention at the most treatable disease stages.

Current commercial MCD tests demonstrate significant limitations for early-stage detection. Grail’s Galleri test shows a sensitivity of only 16.8% for Stage I cancers across all cancer types, while demonstrating a higher sensitivity for advanced stages (90% for Stage IV) [9]. Similarly, Thrive’s CancerSEEK achieves a 43% sensitivity for Stage I disease [11]. The 100% Stage I sensitivity achieved in this study represents a substantial improvement over existing technologies.

The tissue-of-origin accuracy of 98% also exceeds current commercial offerings. Grail’s Galleri test provides a definitive TOO identification in approximately 93% of cases when cancer is detected [9], while CancerSEEK demonstrates a lower TOO accuracy, particularly for certain cancer types [11]. The high TOO accuracy achieved in this study would minimize downstream diagnostic testing requirements, reducing patient anxiety, healthcare costs, and diagnostic delays, while accelerating disease confirmation and subsequent treatment selection.

Individual cancer-type analysis reveals important performance variations. Breast cancer, representing the largest cohort, achieved a 96.6% specificity with 100% sensitivity, suggesting its potential utility as a confirmatory test following abnormal mammography. This application may significantly reduce unnecessary biopsies, which currently occur in approximately 1.3 million women annually in the United States following false-positive mammograms [1].

Lung cancer performance (96.7% specificity, 100% sensitivity) suggests similar confirmatory testing applications following abnormal LD-CT scans. Given that 33% of LD-CT scans yield false-positive results requiring follow-up evaluation [2], a confirmatory blood test could substantially reduce unnecessary downstream procedures and patient anxiety.

The achievement of 100% specificity for ovarian, colorectal, and pancreatic cancers is particularly significant given the lack of established screening protocols for ovarian and pancreatic cancers. These results suggest potential primary screening applications for high-risk populations or symptomatic individuals.

The protein-based approach offers several theoretical advantages over DNA-based methods. Proteins represent direct functional outputs of cellular processes and are present in higher concentrations than circulating DNA fragments. The measurement of enzymatic activities, particularly kinase activities, provides functional rather than merely quantitative information about cancer biology. Additionally, the assessment of both innate (IgM) and activated (IgG) immune responses to cancer-associated proteins captures the dynamic nature of cancer-immune system interactions.

The simplicity of blood-based testing offers significant advantages for clinical implementation compared to imaging-based screening. Blood collection can be performed in primary care settings without specialized equipment or trained technologists, potentially improving screening access in underserved populations.

Cost considerations favor protein-based approaches over DNA-based methods, as protein assays typically cost USD 50–200 per test compared to USD 1000–3000 for comprehensive ctDNA analysis. This cost differential could facilitate a broader screening implementation and improve cost-effectiveness compared to current screening modalities.

## 5. Conclusions

This study establishes the foundation for a highly effective protein-based multi-cancer early detection test that addresses the critical limitations of current screening methodologies. The achievement of 100% sensitivity across five major cancer types, combined with a 97% overall specificity and 98% tissue-of-origin accuracy, represents a significant advancement in liquid biopsy technology for cancer screening applications.

The exceptional performance for Stage I cancer detection addresses a fundamental requirement for effective screening that existing technologies fail to meet. The high tissue-of-origin accuracy (98%) minimizes downstream testing requirements, potentially reducing healthcare costs, patient anxiety, and diagnostic delays compared to current multi-cancer detection approaches.

These findings suggest that protein-based biomarker approaches may overcome the sensitivity limitations inherent in DNA-based methods while providing superior tissue-of-origin identification. The simplicity and cost-effectiveness of protein-based testing could facilitate a broader screening implementation, particularly in underserved populations with limited access to specialized imaging facilities.

While these results are promising, validation in diverse populations and larger cohorts is essential before clinical implementation. The integration of this technology with existing screening programs could potentially transform cancer detection by enabling early intervention at the most treatable disease stages across multiple cancer types simultaneously.

Several limitations must be acknowledged in interpreting these results. The study cohort was limited to Caucasian participants, requiring validation in diverse populations before broad clinical implementation. Sample sizes for colorectal (*n* = 13), ovarian (*n* = 13), and pancreatic (*n* = 11) cancers were small, resulting in wider confidence intervals for performance metrics. These smaller cohorts require expansion in future validation studies.

The retrospective study design using banked samples may not fully represent prospective screening populations. Future studies should employ prospective enrollment to confirm these findings. Additionally, the comparison group consisted of apparently healthy controls rather than individuals presenting for cancer screening evaluation, who may have symptoms or risk factors that could affect biomarker patterns.

The rule-based classification approach, while effective, may benefit from integration with machine learning algorithms to optimize its performance further as larger datasets become available. The current approach may not capture complex biomarker interactions that advanced algorithms could identify.

Long-term follow-up data are needed to assess clinical outcomes, including interval cancer detection, false-negative rates in practice, and impact on cancer mortality. Additionally, health economic analyses are required to determine the test’s cost-effectiveness compared to current screening strategies.

Validation studies in diverse populations represent the highest priority for future research. These blinded studies should include participants with varying demographic characteristics, comorbidities, and risk factor profiles to ensure broad applicability. Sample sizes should be powered to provide narrow confidence intervals for all cancer types, particularly those with smaller representation in this study.

Longitudinal studies to track biomarker patterns over time could provide insights into cancer development and progression, potentially enabling risk stratification and personalized screening intervals. Additionally, the investigation of biomarker patterns in pre-cancerous conditions could extend the utility of this approach to cancer prevention applications.

## Figures and Tables

**Table 1:** Demographics and disease characteristics.

Category	Description	BR	CRC	LNG	PAN	OVA	Normal
AGE	*n*	61	13	43	11	13	119
	mean	56.33	62.85	65.81	63.18	56.31	58.52
	std	12.504	8.849	8.582	11.241	13.193	7.897
	median	54.00	62.00	65.00	63.00	54.00	57.50
	min	32.00	51.00	45.00	37.00	31.00	46.00
	max	79.00	78.00	76.00	76.00	74.00	75.00
	95% CI	[53–59]	[57–68]	[62–68]	[55–70]	[48–64]	[56–60]
							
Ethnicity	Caucasian	(61, 100)	(13, 100)	(43, 100)	(11, 100)	(13, 100)	(119, 100)
							
Sex	Female	(59, 96.7)	(8, 61.5)	(14, 32.6)	(8,72.7)	(13, 100)	(89, 74.8)
	Male	(2, 0.3)	(5, 38.4)	(29, 67.4)	(3,23.2)		(30, 25.2)
							
Stage	0	(2, 3.3)					
	I	(11, 18.0)	(1, 7.7)		(1, 9.1)		
	IA	(3, 4.9)		(14, 32.6)			
	IB			(17, 39.5)		(1, 7.7)	
	II		(4, 30.8)				
	IIA	(22, 36.0)			(2, 18.2)	(2, 15.4)	
	IIB	(11, 18.0)		(1, 2.3)	(1, 9.1)		
	III		(4, 30.8)		(4, 36.3)		
	IIIA	(7, 11.5)		(4, 9.3)		(4, 30.8)	
	IIIB			(1, 2.3)			
	IIIC	(1, 1.6)				(5, 38.5)	
	IV		(4, 30.8)	(3, 7.0)	(3, 27.2)	(1, 7.7)	
	N/A	(4, 6.5)		(3, 7.0)			

Note: BR = Breast, CRC = Colorectal, LNG = Lung, PAN = Pancreatic, OVA = Ovarian. N/A = not available.

**Table 2:** Biomarker values relative to Normal.

							Female	
		BR	CRC	LNG	PAN	OVA	Normal	Normal
xPKA	mean	50	67	35	25	23	75	75
	median	40	65	30	25	20	60	65
Kinase 1	mean	85	210	75	50	60	120	115
	median	75	210	70	50	75	80	80
Kinase 2	mean	80	40	60	85	45	75	76
	median	75	38	40	75	45	70	70
Kinase 3	mean	30	30	50	20	25	55	55
	median	15	20	35	15	20	35	35
Kinase 4	mean	175	275	110	75	90	215	210
	median	170	275	110	75	75	175	175
Anti-1	mean	0.25	0.25	0.4	0.2	0.25	0.85	0.8
	median	0.15	0.2	0.3	0.15	0.2	0.25	0.3
Anti-2	mean	0.2	0.65	0.7	0.2	0.25	0.8	0.75
	median	0.1	0.65	0.5	0.1	0.2	0.3	0.35
Anti-3	mean	4.8	1.3	2.1	2	1.3	5.5	4.5
	median	0.45	0.35	0.5	0.85	0.45	0.6	0.6
Anti-4	mean	0.2	0.35	0.5	0.15	0.15	0.5	0.5
	median	0.1	0.2	0.35	0.1	0.1	0.3	0.35
Anti-5	mean	0.85	1.05	0.5	1.25	0.7	0.8	0.8
	median	0.55	0.75	0.5	0.7	0.7	0.5	0.5
Anti-6	mean	1.3	1.5	1.5	1.3	1.25	1.25	1.25
	median	0.9	1.5	1	0.85	1.4	0.8	0.8
Anti-7	mean	4.8	1.3	2.1	2	1.3	5.4	4.5
	median	0.45	0.35	0.5	0.8	0.45	0.6	0.6
Anti-8	mean	0.25	0.35	0.5	0.15	0.2	0.6	0.6
	median	0.1	0.2	0.35	0.2	0.15	0.35	0.4
Anti-9	mean	0.6	1.25	1.3	1.2	0.65	1.1	0.9
	median	0.5	0.8	0.8	0.6	0.65	0.6	0.6
Anti-10	mean	1.3	1.6	1.6	1.2	1.25	1.5	1.5
	median	1	1.5	1.2	0.8	1.2	1	1
Anti-11	mean	1.25	1.6	1.75	1.3	1.3	1.5	1.5
	median	1	1.5	1.35	0.9	1.4	1	1

**Table 3:** Summary of sensitivity, specificity, accuracy, PPV, and NPV.

	BR	CRC	LNG	PAN	OVA
Cancer	*N* = 61	*N* = 13	*N* = 43	*N* = 11	*N* = 13
Normal	*N* = 89	*N* = 119	*N* = 119	*N* = 119	*N* = 89
Sensitivity	100%	100%	100%	100%	100%
Specificity	97%	99%	97%	100%	100%
Accuracy	98%	99%	98%	100%	100%
PPV	64%		26%		
NPV	100%		100%		

**Table 4:** 95% Confidence Intervals.

	Sensitivity	Specificity	TOO
Breast	94–100%	97–100%	92–99%
Lung	94–100%	95–100%	89–98%
Colorectal	75–100%	97–100%	98–100%
Ovarian	75–100%	96–100%	98–100%
Pancreatic	72–100%	97–100%	98–100%

## Data Availability

The anonymized original data presented in the study are openly available in the dataset named Cancer biomarkers at https://dataverse.harvard.edu/dataset.xhtml?persistentId=doi:10.7910/DVN/1MAXJC and published in Harvard Dataverse at https://dataverse.harvard.edu/dataverse/harvard. URL accessed on 25 July 2025.
