# Correction: Held et al. A Protein-Based Blood Test for Multi-Cancer Diagnostics. *Biomedicines* 2025, *13*, 2510

**DOI:** 10.3390/biomedicines14010132

**Published:** 2026-01-09

**Authors:** Douglas Held, Steven Bolland, Robert Freese, Robert Puskas

**Affiliations:** 1 Traxxsson, LLC, Saint Louis, MO 63021, USA; 2 Definitive Diagnostics, New York, NY 10021, USA

**Error in Tables**

In the original publication, there was a mistake in Table 1 as published [1]. The male breast cancer percentage should be 3.3% instead of 0.3%. The corrected Table 1 appears below.

In the original publication, there was a mistake in Table 4 as published [1]. Breast specificity should be corrected to 90–99% of 95% CI. Lung specificity should be corrected to 92–99% of 95% CI. The corrected Table 4 appears below.

The authors state that the scientific conclusions are unaffected. This correction was approved by the Academic Editor. The original publication has also been updated.

## Figures and Tables

**Table 1:** Demographics and disease characteristics.

Category	Description	BR	CRC	LNG	PAN	OVA	Normal
AGE	*n*	61	13	43	11	13	119
	mean	56.33	62.85	65.81	63.18	56.31	58.52
	std	12.504	8.849	8.582	11.241	13.193	7.897
	median	54.00	62.00	65.00	63.00	54.00	57.50
	min	32.00	51.00	45.00	37.00	31.00	46.00
	max	79.00	78.00	76.00	76.00	74.00	75.00
	95% CI	[53–59]	[57–68]	[62–68]	[55–70]	[48–64]	[56–60]
Ethnicity	Caucasian	(61, 100)	(13, 100)	(43, 100)	(11, 100)	(13, 100)	(119, 100)
Sex	Female	(59, 96.7)	(8, 61.5)	(14, 32.6)	(8,72.7)	(13, 100)	(89, 74.8)
	Male	(2, 3.3)	(5, 38.4)	(29, 67.4)	(3,23.2)		(30, 25.2)
Stage	0	(2, 3.3)					
	I	(11, 18.0)	(1, 7.7)		(1, 9.1)		
	IA	(3, 4.9)		(14, 32.6)			
	IB			(17, 39.5)		(1, 7.7)	
	II		(4, 30.8)				
	IIA	(22, 36.0)			(2, 18.2)	(2, 15.4)	
	IIB	(11, 18.0)		(1, 2.3)	(1, 9.1)		
	III		(4, 30.8)		(4, 36.3)		
	IIIA	(7, 11.5)		(4, 9.3)		(4, 30.8)	
	IIIB			(1, 2.3)			
	IIIC	(1, 1.6)				(5, 38.5)	
	IV		(4, 30.8)	(3, 7.0)	(3, 27.2)	(1, 7.7)	
	N/A	(4, 6.5)		(3, 7.0)			

Note: BR = Breast, CRC = Colorectal, LNG = Lung, PAN = Pancreatic, OVA = Ovarian, N/A = not available.

**Table 4:** 95% Confidence Intervals.

	Sensitivity	Specificity	TOO
Breast	94–100%	90–99%	92–99%
Lung	94–100%	92–99%	89–98%
Colorectal	75–100%	97–100%	98–100%
Ovarian	75–100%	96–100%	98–100%
Pancreatic	72–100%	97–100%	98–100%

## References

[1] Held D., Bolland S., Freese R., Puskas R. (2025). A Protein-Based Blood Test for Multi-Cancer Diagnostics. Biomedicines.

